# Reference Gene Validation for Quantitative RT-PCR during Biotic and Abiotic Stresses in *Vitis vinifera*

**DOI:** 10.1371/journal.pone.0111399

**Published:** 2014-10-23

**Authors:** Alexandre Filipe Borges, Catarina Fonseca, Ricardo Boavida Ferreira, Ana Maria Lourenço, Sara Monteiro

**Affiliations:** 1 Disease & Stress Biology Laboratory, Instituto de Tecnologia Química e Biológica, Universidade Nova de Lisboa, Oeiras, Portugal; 2 Disease & Stress Biology Laboratory, Instituto Superior de Agronomia, Universidade de Lisboa, Lisboa, Portugal; 3 REQUIMTE, Departamento de Química, Faculdade de Ciências e Tecnologia, Universidade Nova de Lisboa, Caparica, Portugal; Institute for Sustainable Plant Protection, C.N.R., Italy

## Abstract

Grapevine is one of the most cultivated fruit crop worldwide with *Vitis vinifera* being the species with the highest economical importance. Being highly susceptible to fungal pathogens and increasingly affected by environmental factors, it has become an important agricultural research area, where gene expression analysis plays a fundamental role. Quantitative reverse transcription polymerase chain reaction (*q*RT-PCR) is currently amongst the most powerful techniques to perform gene expression studies. Nevertheless, accurate gene expression quantification strongly relies on appropriate reference gene selection for sample normalization. Concerning *V. vinifera*, limited information still exists as for which genes are the most suitable to be used as reference under particular experimental conditions. In this work, seven candidate genes were investigated for their stability in grapevine samples referring to four distinct stresses (*Erysiphe necator*, wounding and UV-C irradiation in leaves and *Phaeomoniella chlamydospora* colonization in wood). The expression stability was evaluated using geNorm, NormFinder and BestKeeper. In all cases, full agreement was not observed for the three methods. To provide comprehensive rankings integrating the three different programs, for each treatment, a consensus ranking was created using a non-weighted unsupervised rank aggregation method. According to the last, the three most suitable reference genes to be used in grapevine leaves, regardless of the stress, are *UBC*, *VAG* and *PEP*. For the *P. chlamydospora* treatment, *EF1*, *CYP* and *UBC* were the best scoring genes. Acquaintance of the most suitable reference genes to be used in grapevine samples can contribute for accurate gene expression quantification in forthcoming studies.

## Introduction

Grapevine is one of the most cultivated fruit crop worldwide with *Vitis vinifera* being the species with the highest economical importance due to the high quality standards of its berries. Nonetheless, it is also the most susceptible *Vitis* species to fungal pathogens which can have devastating consequences in the vineyards [1], [2]. The associated crop losses, together with the elevated financial and environmental costs to control these diseases, have made *Vitis vinifera* and its pathogens an increasingly important research area. Common approaches to study the corresponding pathosystems often involve, at least at one point, transcriptomic analysis and gene expression quantification [3]–[5]. Among the several existing techniques to analyze mRNA levels, *q*RT-PCR is currently the most widely used due to its high sensitivity and reproducibility [6]. However, accurate gene expression quantification strongly relies on appropriate reference gene selection for sample normalization [7]–[9]. Though this requirement has always been an important criterion for gene expression quantification studies, during the early stages of *q*PCR expansion and data analysis development, reference gene selection was rather based on assumptions more than evidence on expression stability. As consequence, several studies might have been conducted using unsuitable or unvalidated reference genes [10], [11]. Recent awareness regarding this matter has lead to an increasing number of studies whose main objective is to evaluate the expression stability of candidate genes for normalization in *q*PCR analysis [12]–[15]. Under the most diverse experimental conditions, including a variety of organisms or tissues and a multitude of biotic and abiotic stimulus, such analysis can provide a valuable tool for accurate gene expression quantification in forthcoming studies. To assess the gene expression stability of potential reference genes, several programs and statistical algorithms have been developed, facilitating the analysis and selection of suitable reference genes for the desired experimental condition. geNorm, NormFinder and BestKeeper are among the most widely used algorithms [7], [16], [17]. With respect to *V. vinifera*, a non-model organism, some studies have already been conducted in order to evaluate and validate *q*RT-PCR reference genes to be used under distinct physiological and pathological conditions. Those comprise specific aspects of *V. vinifera* such as genotypes, organs, developmental stages, abiotic stresses and biotic stresses: (i) berries at different stages of development [18]; (ii) leaves subjected to two different abiotic stresses: drought and temperature [19]; (iii) berries from different genotypes at distinct phenological stages, treated or not with gibberellic acid [20]; (iv) leaves at late stages of infection with *Plasmopara viticola* and berries at late stages of infection with *Botrytis cin*erea [11]; (v) two genotypes of *V. vinifera* leaves exhibiting differential sensitivity to *Plasmopara viticola* at the early stages of infection with the oomycete [21]; (vi) leaves infected with *Plasmopara viticola* at late stages of infection [22]. Also, a previous work performed in our laboratory evaluates reference genes to be used in leaf samples at late stages of infection with *Erysiphe necator* [23].

Despite the valuable contribution of the previous works, other stress conditions of great importance to plant studies, such as the abiotic stresses caused by wounding or UV-C irradiation, remain to be addressed for this species [24]. Due to their sessile nature, plants are permanently exposed to a wide range of structurally damaging agents which include environmental stresses such as wind, rain or hail, and herbivore attack. Wound occurrence is inevitable and, besides compromising the physical structure of the plant, it constitutes a potential infection site for pathogens [24], [25]. To cope with this dual threat, plants might have evolved to integrate both wounding and pathogen response. In fact, some studies have already demonstrated that both stresses can share common signaling pathways and, moreover, regulate the same stress responsive genes. For this reason, wound response stands as an important area of interest in plant studies [26], [27]. As for the UV-C stimulus, though such short wavelength radiation is not likely to reach the ground, it has been shown, in several species, that UV-C irradiation can enhance host resistance to pathogens [28]. Also, in the particular case of *V. vinifera*, UV-C was shown to induce the accumulation of several phenolic defense-related compounds, including resveratrol and other stilbene derivatives. Thus, UV-C irradiation constitutes a practical experimental model to study plant defense responses [29], [30].

Concerning the biotic stresses affecting grapevine, besides *P. viticola* and *E. necator*, also *Phaeomoniella chlamydospora* stands amongst the most concerning pathogens for viticulture. While the former oomycetes infect the leaves and berries of the host, the second is a wood colonizing fungus known to participate in the esca disease complex. Taken together, these diseases account for huge economical losses and, therefore, represent priority research areas [21], [31]–[33]. Yet, to our knowledge, no reference gene stability studies have been performed for the host during *P. chlamydospora* infection.

Given the relevance of gene expression studies and the imperative need to use suitable reference genes for sample normalization in *q*PCR analyses, the previous considerations prompted us to determine the most stable reference genes, among seven candidates, during three distinct stresses: *P. chlamydospora* trunk infection, leaf wounding and UV-C irradiation. Moreover, we complemented our previous stability analysis concerning *V. vinifera-E. necator* interaction for which only geNorm software had been used [23]. In view of the different stresses known to occur in plants, the selection of the stimuli addressed in the present work aims at increasing the existing information concerning suitable grapevine reference genes to be used in future gene expression studies.

## Materials and Methods

### Plant material and growth conditions

Grapevine (*Vitis vinifera* L., cultivar Touriga Nacional) cuttings used in the experiment were collected from Centro Experimental de Pegões, Portugal and subjected to heartwood disease screening through microbiology assays. Sample collection was gently and duly authorized by Dr. Antero Martins, Associação Portuguesa para a Diversidade da Videira (PORVID), Portugal. The microbiologic screening was performed using the bottom of the cuttings. Thin wood slices were removed from each cutting, surface-sterilized (ethanol, flame and sodium hypochlorite) and then placed in 0.03% (w/v) chloramphenicol-containing PDA medium (five slices per cutting). The plates were incubated at room temperature for a maximum period of one month, during which morphological identification of the microorganisms present in the wood was performed. Diseased cuttings were discarded.

Healthy *V. vinifera* cuttings, with three buds each, were rooted in water and then transferred to soil (1 L pot per plant). Plants were maintained in a growth chamber at 25°C with a photoperiod of 16 h (480 µmol.m**^−2^**.s**^−1^**). After one month of acclimatization period, whole plants or detached leaves were subjected to the different treatments.

### Plant treatments

For the powdery mildew (PM) treatment, all plants within the same growth chamber were simultaneously inoculated with *E. necator* by direct contact with naturally infected grapevine leaves. The primary inoculum was collected from a vineyard in Instituto Superior de Agronomia, Lisbon, Portugal and passed to a set of grapevines in greenhouse which provided the experimental inoculum source. Plants were allowed to grow with generalized powdery mildew infection for 30 days prior to sample collection. Fully expanded leaves (fourth and fifth positions from the tip of each shoot) with and without *E. necator* infection symptoms (visible mycelia on the upper leaf surface) were harvested.

For the wounding treatment, fully expanded leaves were cut using a sterile razor blade. Each leaf was subjected to six 1 cm-long cuts and collected 24 h after the treatment. Control samples were left untreated and maintained under the same conditions.

For the UV irradiation treatment, leaves were detached and their undersides were exposed to UV-C radiation (Philips TUV 30 W, 92 µW cm-2 at 253 and 7 nm) at a distance of 15 cm from the source during 10 min. Following irradiation, treated and control samples were incubated in a dark wet chamber at room temperature for 48 h.

For the *Phaeomoniella chlamydospora* treatment, a pure fungal isolate was obtained from CBS (CBS 239.74) and propagated in PDA medium at 23°C in dark. Inoculation was performed at the base of the primary shoot by removing a small section of the bark with a scalpel and placing a 5 mm inoculation plug (cut from the actively growing margin of the colony) into the wound (mycelium side down). Each wound was then be covered with moist cotton wool and sealed with parafilm. The same procedure was followed for negative control plants using non inoculated PDA plugs. Plants were maintained under the above described conditions for one week prior to sample collection.

Following the mentioned incubation periods for individual treatments, all samples were harvested and immediately frozen in liquid nitrogen. Biological replicates for all treatments and corresponding controls were created by pooling either four leaves or two 5 cm-long stem sections per sample.

### RNA extraction and cDNA synthesis

Total RNA extraction was performed using the Rapid CTAB (hexadecyltrimethyl ammonium bromide) method, especially suited for high phenolic content material, adapted as follows [34]. Biological samples were ground in liquid nitrogen, homogenized at approximately 150 mg per mL in extraction buffer (2% (w/v) CTAB, 2.5% (w/v) polyvinylpoly-pyrrolidone, 2 M NaCl, 100 mM Tris-HCl pH 8.0, 25 mM ethylenediaminetetraacetic acid (EDTA), 2% (v/v) β-mercaptoethanol) and incubated at 65°C for 10 min. Samples were extracted twice with one volume of chloroform:isoamylic alcohol (24∶1, v/v) and centrifuged at 12,000 *g* during 10 min at 4°C. The recovered aqueous phase was supplemented with ¼ volume of 10 M LiCl and incubated during 30 min at 4°C. RNA was collected by centrifugation at 21,000 *g*, 4°C during 20 min, and resupended in 500 µL of pre-warmed (65°C) SSTE buffer (0.5% w/v sodium dodecyl sulfate (SDS), 1 M NaCl, 10 mM Tris-HCl pH 8.0, 1 mM EDTA). Samples were again extracted with one volume of chloroform:isoamylic alcohol (24∶1, v/v) followed by centrifugation at 12,000 *g* during 10 min. The recovered supernatant was supplemented with 0.7 volumes of cold isopropanol and immediately centrifuged at 21,000 *g*, 4°C during 15 min. RNA *pellet* was washed with 70% (v/v) ethanol and resuspended in water. Prior to reverse transcription, samples were treated with RQ1 RNase-Free Dnase (Promega) according to the manufacturer's protocol.

All samples were reverse transcribed using ThermoScript RT-PCR System (Invitrogen) as described by the manufacturer. cDNA was synthesized from 1.5 µg of total RNA and oligo(dT)**_20_** primers. RT reactions were carried at 55°C for 60 min.

### Primer design and qPCR

PCR primers were designed with Beacon Designer software (Premier Biosoft International) to target amplicons between 80 and 300 bps. Amplification specificity was first assessed though Primer-BLAST (http://www.ncbi.nlm.nih.gov/tools/primer-blast/) using *V. vinifera* database as template. qPCR was performed with iQ SYBR Green supermix (Bio-Rad) using iCycler equipment (Bio-Rad). Prior to use, cDNA samples were diluted to 50 ng/µL. Reaction mixtures (20 µL) were prepared according to the following: 5 µL of the diluted template, 1 µL primer mix (10 µM each), 10 µL iQ SYBR Green supermix (Bio-Rad), 4 µL H_2_O. Thermal cycling was composed of an initial denaturation step for 3 min at 95°C, 40 cycles at 95°C for 10 s, 55°C for 30 s and 72°C for 30 s. All reactions were performed in triplicate and amplification specificity was confirmed through melting curve analysis.

### Data analysis

Raw data (i.e. not baseline-corrected) belonging to each individual amplification curve was imported from iQ5 into LinRegPCR software (version 11.0) for baseline and PCR efficiency estimation. Log-linear phases were automatically determined containing four to six points with the highest correlation coefficient. According to the obtained linear regressions, individual PCR amplification efficiencies were calculated. Student's *t*-test was used to compare amplification efficiencies of each amplicon between treated samples and corresponding controls of the same treatment (*P*<0.05). Since no differences were observed, mean efficiencies for each amplicon within each treatment were used for subsequent analysis. *Ct* values were retrieved using a fluorescence threshold defined within a common window-of-linearity (WoL) for each dataset.

To evaluate the expression stability of the selected candidate genes for the different stimulus, three different Visual Basic Application (VBA) applets for Microsoft Excel were used: geNorm v.3.5 [7], NormFinder v. 0953 [16] and BestKeeper [17]. Input file creation and subsequent data analysis was performed according to the corresponding manuals. For both GeNorm and NormFinder software, *Ct* values were transformed into relative quantities (amplification efficiency corrected) using the lowest *Ct* sample as calibrator. For BestKeeper analysis, raw *Ct* values as well as PCR amplification efficiencies were directly inserted into the software.

Consensus ranks, integrating the results of the different algorithms, were generated using a non-weighted unsupervised rank aggregation method. Data analysis was carried out using the RankAggreg v. 0.4–3 package [35] for R. RankAggreg input was a matrix of rank-ordered genes according to the different algorithms used. Comprehensive ranks were obtained from the calculated Spearman footrule distances and the Cross-Entropy Monte Carlo algorithm.

## Results and Discussion

### Expression profile of candidate reference genes

**Figure 1:**
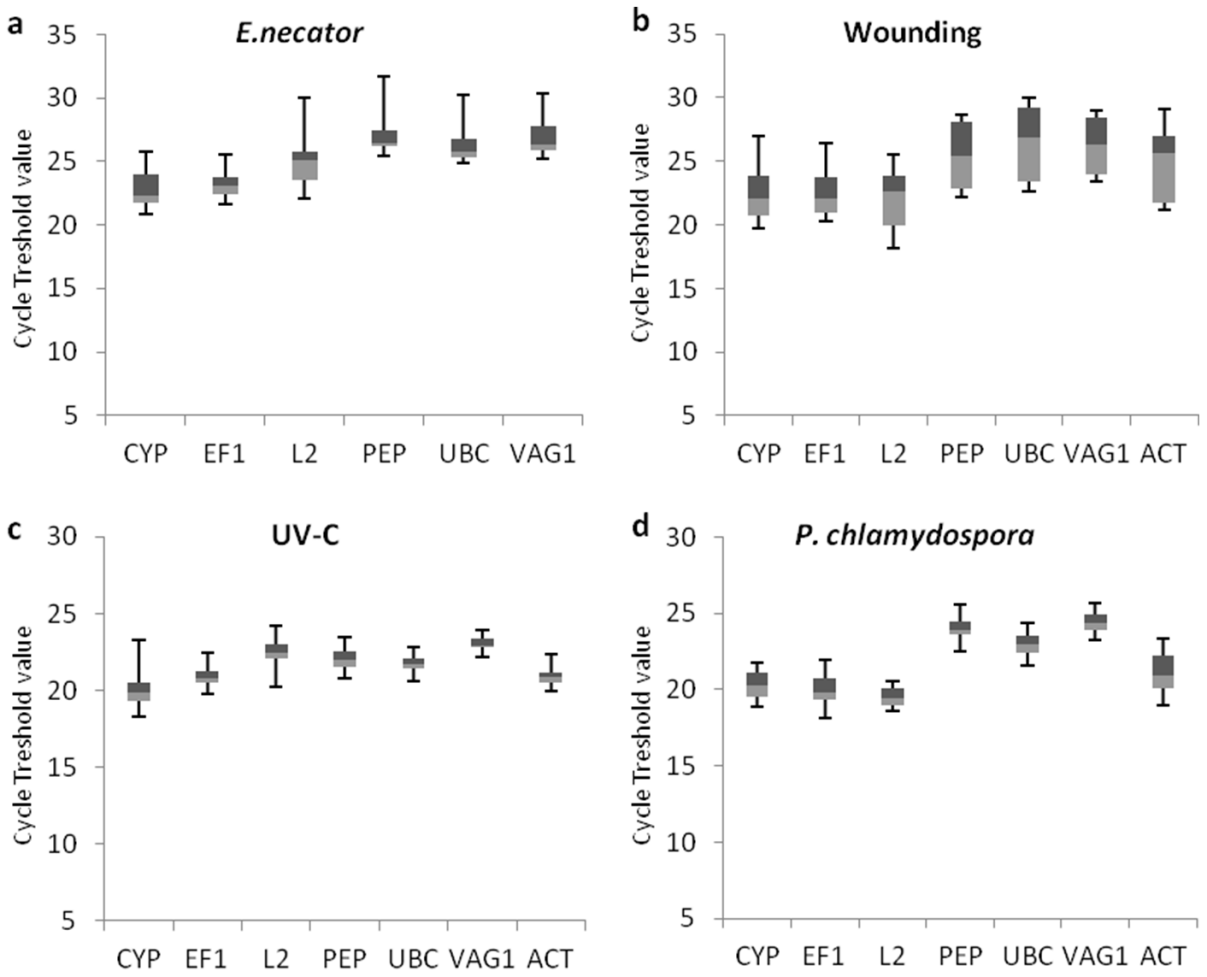
Expression profile of candidate normalization genes in grapevine samples during (a) leaf infection with *E. necator*, (b) leaf wounding, (c) leaf irradiation with UV-C and (d) wood infection with *P. chlamydospora*. Absolute *Ct* values for each treatment and the corresponding controls were combined. Each sample group comprises 5 to 7 biological replicates. The boxes indicate the 25^th^ and 75^th^ percentiles. Lines within the boxes represent the median. Maximum and minimum values are represented by wiskers.

Reference gene validation for qRT-PCR expression studies has become a fundamental requisite for reliable quantification results. To provide information regarding potential reference genes for future use in *q*RT-PCR studies involving *V. vinifera*, we decided to evaluate the expression stability of a set of commonly used housekeeping genes during four distinct stimuli comprising biotic and abiotic stresses [36]–[38]. These include powdery mildew infection, mechanical wounding and UV-C irradiation in leaves and xylem colonization with *P. chlamydospora* in woody tissues. Treatment selection was based on its potential application for future gene expression studies in grapevine. Candidate genes were selected taking into consideration either its frequent use as sample normalizers or their repeated participation in similar studies. To avoid co-regulation events, candidate genes were also required to belong to distinct metabolic pathways. Accordingly, the selected candidates for this study were cyclophilin (*CYP*), elongation factor 1 α (*EF1*), ribosomal protein L2 (*L2*), phosphoenolpiruvate carboxylase (*PEP*), ubiquitin conjugating enzyme (*UBC*), vacuolar ATPase subunit G (*VAG*) and actin (*ACT*) [36]–[42]. Following PCR amplification, a general overview of the expression profile and relative abundance of each candidate gene was obtained by plotting the *Ct* values obtained for all samples (control and treatment) under the different conditions studied (Figure 1). *ACT* expression stability upon *E. necator* infection (Figure 1a) was not assessed as it was not considered as a candidate in our previous study [23].

All genes displayed moderate expression levels with mean *Ct* values ranging from 19.7 to 27.2 for *L2* and *PEP* during *P. chlamydospora* and *E. necator* interaction, respectively. Minimum *Ct* values, meaning higher abundance, were observed for *EF1* (18.1) during *P. chlamydospora* interaction, whilst *PEP* displayed the highest *Ct* (31.6) for *E. necator* treatment. Overall, gene expression variation across samples within each treatment ranged from 1.9 to 7.9 *Ct*s with the highest expression fluctuations being observed for the wounding experiment. Though preliminary information can be obtained through absolute *Ct* analysis, to correctly assess the expression stability of candidate genes, raw amplification data must be first linearized. This was carried out by converting the *Ct* values into relative quantities which were normalized to the sample with the lowest *Ct*.

### Primer pair amplification efficiencies

To perform data linearization, PCR amplification efficiencies (*E*) must be taken into consideration, preventing significant bias from being introduced in the generated results [43], [44]. *E* values were estimated using the absolute fluorescence increase method (Table 1) [44], [45]. LinRegPCR software (version 11.0), developed by Ruijter *et al*., 2009, was used to individually analyze each sample and determine amplification efficiencies based on a proper baseline correction. Considering that *E* value for one primer pair might differ among stimuli and, moreover, between control and treated samples in the same stimulus, we separately analyzed each sample group. Though amplification efficiencies for each gene may vary depending on the treatment, no differences (p<0.05) were observed between control and treated samples of the same stimulus. Thus, mean *E* values (Table 1) were used for subsequent analysis.

**Table 1:** Candidate genes and primer pairs for *q*RT-PCR normalization in grapevine samples.

					PCR amplification Efficiency (*E* %)			
Gene	Accession number	Primer	Sequence 5′->3′	Amplicon lenght (bp)	PM^a^	Wound	UV-C	Pch^c^
*CYP*	ES880796	Fw	ACAGCCAAGACCTCGTG	138	78.8	91.2	91.1	78.3
		Rv	GCCTTCACTGACCACAAC					
*EF1*	GU585871.1	Fw	GAACTGGGTGCTTGATAGGC	164	86.7	93.1	91.4	77.8
		Rv	AACCAAAATATCCGGAGTAAAAGA					
*L2*	AJ441290.2	Fw	TCTACTTCAACCGATATGC	199	92.3	93.7	96.3	83.3
		Rv	CCACCTGTCCGACTG					
*PEP*	AF236126.1	Fw	CCTCCTCCTCCAGATTGC	198	89.6	94.0	97.8	82.9
		Rv	GGCTTGCTTGATTCCATTATC					
*UBC*	EE253706	Fw	CATAAGGGCTATCAGGAGGAC	161	87.2	92.7	94.8	83.4
		Rv	TGGCGGTCGGAGTTAGG					
*VAG*	XM_002281110.1	Fw	TTGCCTGTGTCTCTTGTTC	174	91.8	92.3	99.1	84.0
		Rv	TCAATGCTGCCAGAAGTG					
*ACT*	XM_002282480	Fw	GACTACCTACAACTCCATCAT	113	b	94.2	92.6	82.5
		Rv	TCATTCTGTCAGCAATACCA					
*PAL* d	XM_002268220	Fw	TTCCGAACCGAATCAAGG	193	b	90.2	91.5	b
		Rv	GGAGCACCGTTCCAAGC					

a Powdery mildew (*Erysiphe necator*).

b Not determined.

c *Phaeomoniella chlamydospora*.

d Responsive gene used for differential expression quantification.

### Reference gene expression stability

Following baseline estimation and amplification curve analysis for all *q*PCR reactions, the statistical analysis to evaluate the expression stability of the candidate genes was performed using three different programs: geNorm [7], NormFinder [16] and BestKeeper [17]. Though all aim to determine which candidate genes are the most stable under certain conditions, they run under different algorithms and mathematical models. Therefore, the stability ranking of the putative reference genes might differ depending on the program used [46]. GeNorm analysis relies on the intuitive principle that the expression ratio of two ideal reference genes should always remain constant across all samples. Accordingly, it calculates a gene expression stability measure (M) based on the average pairwise expression ratio between each gene and the remaining candidates. Lower M values are indicative of higher stability. The main drawback of geNorm, and consequently one of the most important criteria to be aware of, is that candidate genes must not be co-regulated. This would introduce significant bias as identically regulated genes tend to be top ranked in geNorm even if their expression levels fluctuate considerably among samples [7]. On the other hand, NormFinder analysis, a model-based variance estimation method, displays less sensitivity to co-regulation events. Expression stability of candidate genes is evaluated according to their overall expression variation among the sample set. For each of the analyzed genes, NormFinder calculates a stability value (SV) according to which a ranking is generated. Similarly to geNorm, a lower SV value is indicative of higher stability [16]. The third and last tool adopted to assess the gene expression stability was BestKeeper software. Unlike the previous methods, input data for this software consists of raw *Ct* values instead of relative quantities. Nevertheless, amplification efficiencies are also considered. The expression variability is assessed through coefficient of variance and standard deviation analysis. The software calculates a “BestKeeper index” referring to each sample and compares the candidate genes based on their pairwise correlation with this index value. Candidates displaying a higher Pearson's correlation coefficient (*r*) correspond to the most stably expressed [17].

Following our gene expression variation analysis over the four particular experimental conditions, the candidate genes were rank ordered according to the stability parameters calculated by each program (Table 2).

**Table 2:** Grapevine candidate reference gene stability rankings during different treatments according to geNorm, NormFinder and BestKeeper.

Rank	Program					
	geNorm		NormFinder		BestKeeper	
	Gene	M	Gene	SV	Gene	CC
***E. necator***						
1	*UBC*	0.238	*VAG*	0.219	*PEP*	0.953
2	*VAG*	0.238	*CYP*	0.231	*VAG*	0.911
3	*PEP*	0.290	*EF1*	0.237	*CYP*	0.885
4	*CYP*	0.331	*UBC*	0.251	*UBC*	0.878
5	*EF1*	0.402	*PEP*	0.265	*EF1*	0.856
6	*L2*	0.757	*L2*	0.875	*L2*	0.382
**Wounding**						
1	*PEP*	0.287	*PEP*	0.786	*UBC*	0.997
2	*UBC*	0.287	*L2*	0.939	*VAG*	0.996
3	*ACT*	0.366	*CYP*	0.952	*ACT*	0.995
4	*VAG*	0.471	*VAG*	0.961	*CYP*	0.985
5	*L2*	0.531	*EF1*	1.060	*L2*	0.978
6	*CYP*	0.692	*UBC*	1.170	*PEP*	0.977
7	*EF1*	0.776	*ACT*	4.918	*EF1*	0.945
**UV-C**						
1	*UBC*	0.152	*VAG*	0.111	*L2*	0.897
2	*VAG*	0.152	*UBC*	0.151	*UBC*	0.821
3	*PEP*	0.267	*PEP*	0.184	*PEP*	0.800
4	*L2*	0.400	*ACT*	0.352	*VAG*	0.747
5	*ACT*	0.505	*EF1*	0.640	*ACT*	0.519
6	*CYP*	0.661	*CYP*	0.684	*CYP*	0.133
7	*EF1*	0.746	*L2*	0.948	*EF1*	0.050
***P. chlamydospora***						
1	*EF1*	0.282	*EF1*	0.062	*ACT*	0.986
2	*CYP*	0.282	*CYP*	0.068	*EF1*	0.965
3	*VAG*	0.335	*PEP*	0.081	*PEP*	0.955
4	*UBC*	0.362	*UBC*	0.082	*CYP*	0.955
5	*ACT*	0.396	*VAG*	0.090	*UBC*	0.942
6	*PEP*	0.410	*L2*	0.091	*VAG*	0.933
7	*L2*	0.421	*ACT*	0.096	*L2*	0.922

SV, stability value; CC, Pearson coefficient of correlation.

Concerning the long term interaction between grapevine and *E. necator*, a previous work conducted in our laboratory was already performed, in which the most suitable *q*PCR reference genes were reported using the geNorm software [23]. Nevertheless, to provide complementary information regarding this matter, in the present study, we decided to re-evaluate the expression stability of the same candidate genes using also NormFinder and BestKeeper. In addition, since a different PCR efficiency determination method was employed, a new geNorm analysis was also performed. Though different M values were obtained for each gene, the stability ranking was not affected.

As expected, regardless of the experimental condition and similarly to other reference gene evaluation studies, the studied genes performed differently depending on the analysis program used. Therefore, in the absence of an ideal or preferred method, it is not possible to determine the precise candidate genes most stable under each condition. However, in certain cases, a simple overview of the three ranks can reveal particular tendencies. For instance, for the *E. necator* treatment, *VAG* was consistently ranked among the two most stable genes. Yet, *UBC*, whose M value was the same as *VAG*, was ranked fourth according to NormFinder and BestKeeper. Full agreement was observed regarding *L2*, which was the worst ranked gene in all three methods. As for the wounding stimulus, a higher discrepancy is observed among the three methods. While *PEP* displayed the best stability performance when evaluated by geNorm and NormFinder, it was classified as one of the worst genes by BestKeeper. A similar situation occurs for UBC. Despite the significant discrepancies occurring among the ranks generated by the three softwares, one must also be aware that, in some of those cases, the ranks were generated based upon small differences in the stability parameters indicating that the genes involved might possess expression variations very close to each other. As for the UV-C irradiation treatment, a reasonable consistency is observed, where for all methods, both *UBC* and *PEP* are among the three best ranked genes. *CYP* on the other hand, was classified as the second worst gene regardless of the analysis type. For the last treatment addressed in this work, *P. chlamydospora* infection in woody tissues, a clear difference is observed when the stability rankings are compared with the previous treatments. Both *EF1* and *CYP*, which were constantly amongst the worst scoring genes, are, in this case, two of the most stable candidates. Though the biotic stress itself can cause significant gene expression variations, also a considerable effect is expected due to tissue/organ specific metabolism.

### Consensus Stability Rankings

**Figure 2:**
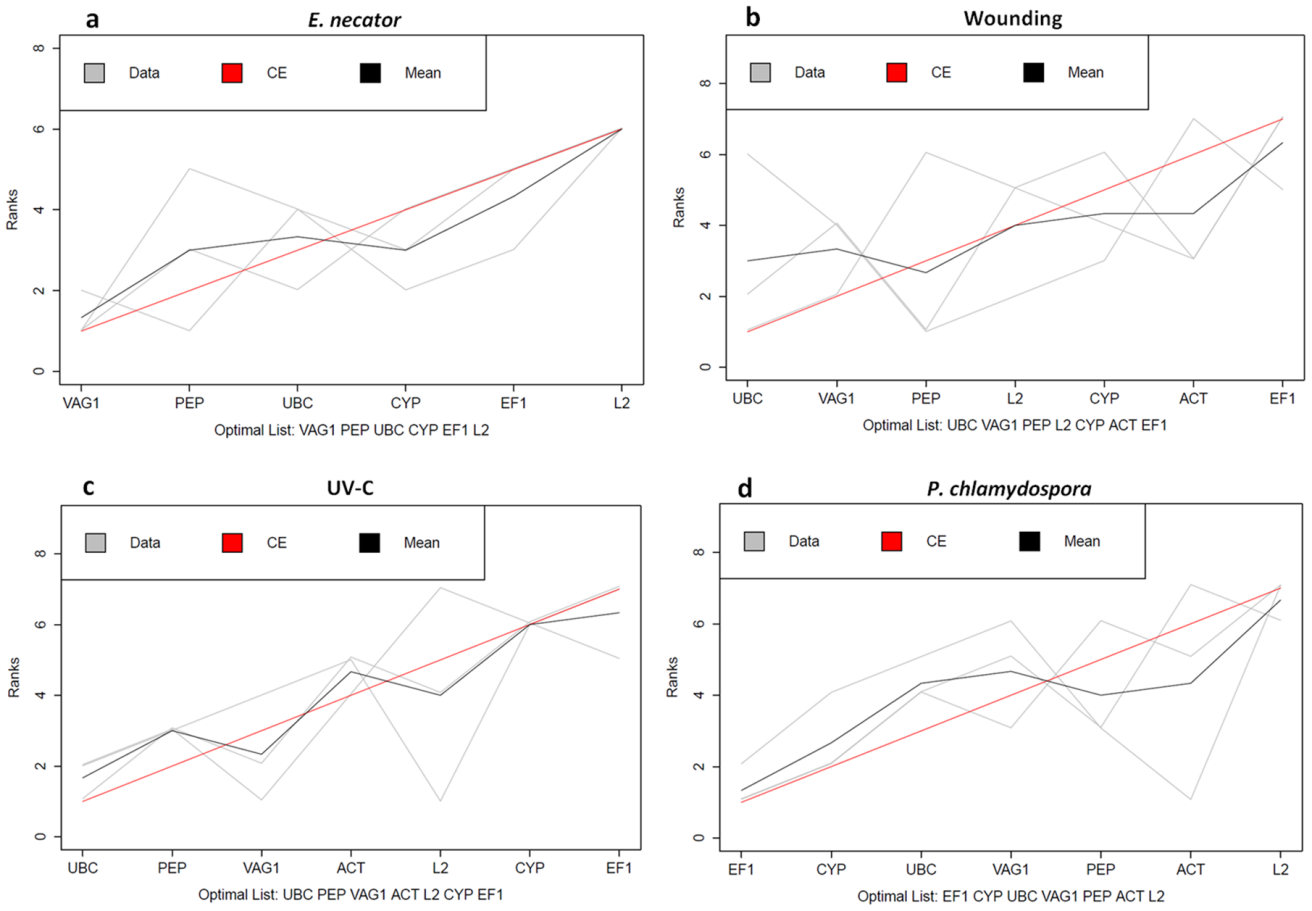
Consensus stability rankings generated by Monte Carlo algorithm for (a) leaf infection with *E. necator*, (b) leaf wounding, (c) leaf irradiation with UV-C, and (d) wood infection with *P. chlamydospora*. RankAggreg (v. 0.4–3) package for R was used to compute Monte Carlo algorithm with the Spearman footrule distances on the rank lists generated by each applet. Individual stability measurements (geNorm, NormFinder or BestKeeper) are shown in grey, average rank positions in black and the computed Monte Carlo model in red.

Considering that each of the previous methods has its own limitations and no agreement exists for which software is the most suitable for expression stability analysis, a common approach to perform these studies often involves the use, comparison and integration of all three methods. Several strategies exist to create a comprehensive stability ranking integrating the results of the three applets. In general, each gene is assigned a certain weight corresponding to the rank obtained for each program (*e.g.* 1-most stable to 7- least stable). Subsequent rank aggregation methodologies are then employed, which can, for instance, rely on straightforward arithmetic and geometric mean of the ranks [47]–[49]. However, in this work a different methodology, suggested by Mallona (2010) and followed by Goulão (2012), was used. The outputs of the different applets were merged by means of a non-weighted unsupervised rank aggregation method using the Cross-Entropy Monte Carlo algorithm. According to the previous method, an optimal stability ranking list for each experimental condition was created (Figure 2).

Overall, the rank aggregation method supports some of our initial observations. When comparing the different optimal lists obtained for each treatment, we can also observe that, for all the three stresses involving grapevine leaves, despite the scoring differences, *UBC*, *VAG* and *PEP* are consistently ranked within the most stable genes. Concerning *P. chlamydospora* treatment, *EF1*, *CYP* and *UBC* are, among all candidates, the most stable reference genes.

For the particular case of *E. necator* infection, an inevitable attempt was made to correlate our results with similar existing studies reporting the most appropriate grapevine normalization genes upon pathogen interaction, namely *Plasmopara viticola*, the causal agent of downy mildew [11], [21], [22]. Though distinct, both pathogens have similar infection mechanisms, lifestyles and colonize the same tissues. In fact, even though different sets of genes were used in each study, a certain degree of accordance can be observed, at least in one of the cases, when some of the same genes are evaluated. In the work conducted by Selim (2012), in which four of the present genes were also evaluated, *UBC* was ranked as the most stably expressed in *P. viticola* infected leaves. In addition, *EF1*, as well as *CYP*, were the two worst ranked candidates. Conversely, in the work developed by Monteiro (2013), also addressing *P. viticola* leaf infection, *EF1* was, at all times, one of top ranked genes.

**Figure 3:**
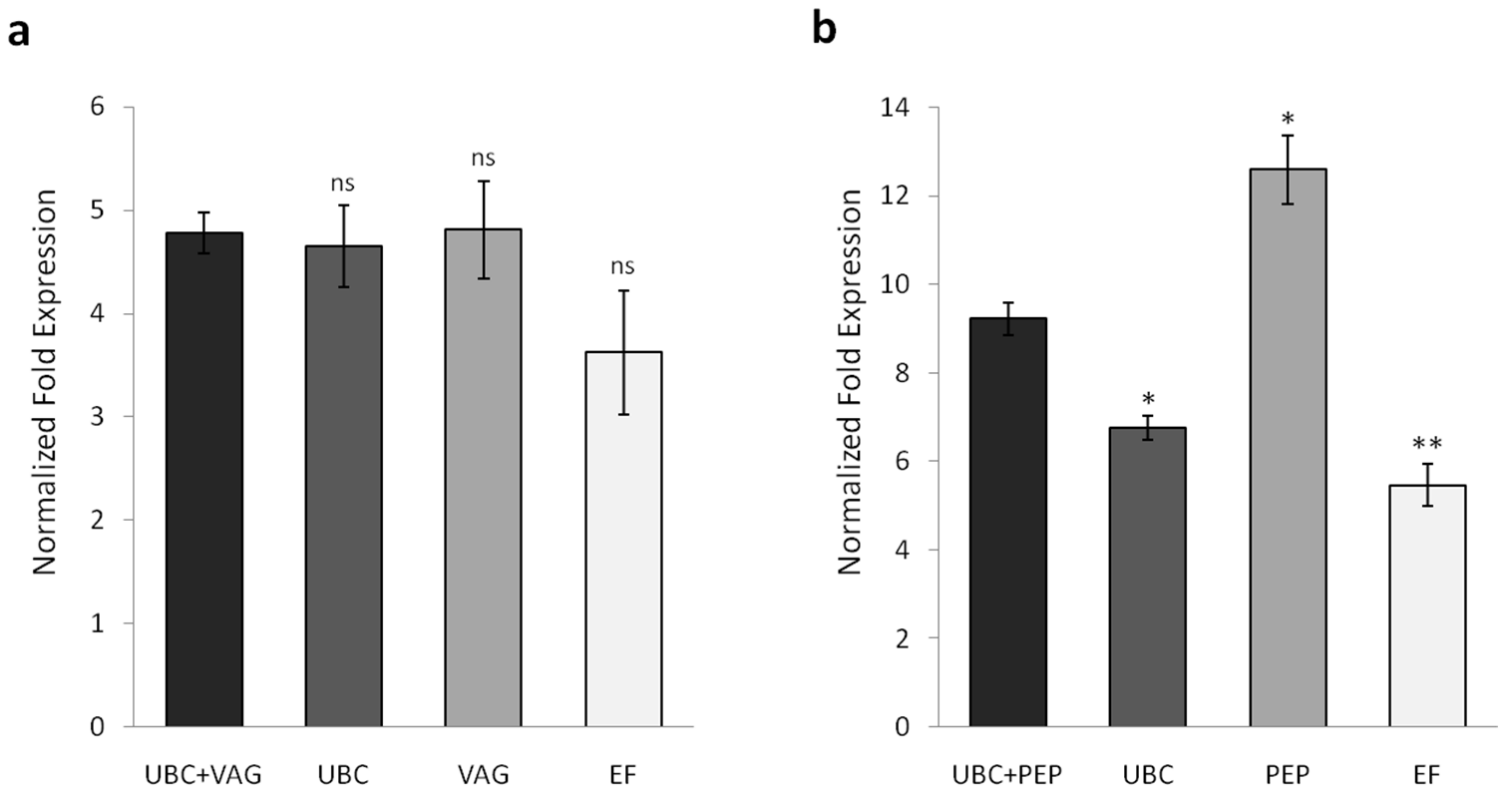
Differential gene expression of *PAL* in grapevine leaves induced by (a) wounding and (b) UV-C irradiation. Relative gene expression quantification was performed using four different normalization factors derived from: the combination of the two top ranked genes, the best ranked gene, the second most stable gene and the worst ranked gene. All values are mean±SD (n = 4). Statistical differences (one-way ANOVA followed by Dunnett's multiple comparison test) to UBC+VAG – wounding or UBC+PEP – UV-C irradiation are marked: ns – not significant (*P*>0.05); * – significant (0.01<*P*<0.05); ** – very significant (0.001<*P*<0.01).

In order to further validate the suitability of the top ranked genes identified in this study, we decided to perform the differential expression quantification of a potentially responsive gene, phenylalanine ammonia lyase (*PAL*), for two of the tested conditions (wounding and UV-C irradiation). Given its extensive characterization and general acceptance as a defense related gene whose expression can be induced by a variety of stresses, *PAL* expression changes caused by the selected stimuli would be predictable to occur [50], [51]. In an attempt to evaluate the potential bias arising due to improper reference gene selection, we calculated the fold change expression of *PAL* using both the best and the worst ranked genes for normalization (Figure 3). For each of the treatments, fold change expression values were determined using different normalization factors (NF) derived from: the combination of the two most stable reference genes, the best ranked gene, the second best gene and the worst ranked gene. As expected, regardless of the treatment, an upregulation of *PAL* was observed. For the wounding experiment (Figure 3a), the calculated expression values using the combined and isolated best genes as NFs (*UBC* and *VAG*) were comparable among themselves, with fold change values of 4.78 (*UBC*+*VAG*), 4.65 (*UBC*) and 4.81 (*VAG*). When using the NF corresponding to the most unstable gene (*EF*), a fold change of 3.62 was obtained. Despite the noticeable difference between the normalization with the worst and best genes, a certain degree of consistency exists within all four quantifications. In fact, the statistical analysis reveals that no significant differences (*P*>0.05) exist when comparing the results obtained from *UBC+VAG* with the remaining. This suggests that the expression of these candidate genes (*UBC*, *VAG* and *EF*), and possibly of all the remaining, was not significantly affected by the experimental wounding stress. Thus, in this case, one could infer that any of the candidates could be used for sample normalization without major bias being introduced. On the other hand, for the UV-C treatment (Figure 3b), larger discrepancies can be observed among the evaluated candidates. *PAL* gene expression normalized to the combination of the two best ranked genes (*UBC* and *PEP*) indicated a 9.22 fold change. However, when normalized for each of the candidates individually, the calculated upregulation was 6.77 (*UBC*), 12.59 (*PEP*) and 5.47 (*EF*). Such accentuated differences highlight not only the importance of selecting the most appropriate reference genes for each experimental conditions, but also the necessity to use multiple genes for sample normalization.

Taken together, besides supporting the already accepted idea that no universal reference genes exist, our results provide information regarding the suitability of potential *q*RT-PCR reference genes to be used in grapevine samples for distinct biotic and abiotic stresses. Such knowledge might prove useful in transcriptomic studies contributing for accurate gene expression quantification.

## References

[1] Iandolino A, Nobuta K, da Silva F, Cook D, Meyers B (2008) Comparative expression profiling in grape (Vitis vinifera) berries derived from frequency analysis of ESTs and MPSS signatures. BMC Plant Biology 8: 53. 18474095 10.1186/1471-2229-8-53 PMC2423195

[2] Ferreira RB, Monteiro SS, Piçarra-Pereira MA, Teixeira AR (2004) Engineering grapevine for increased resistance to fungal pathogens without compromising wine stability. Trends in Biotechnology 22: 168–173. 15038921 10.1016/j.tibtech.2004.02.001

[3] Lima MM, Ferreres F, Dias AP (2012) Response of Vitis vinifera cell cultures to Phaeomoniella chlamydospora: changes in phenolic production, oxidative state and expression of defence-related genes. European Journal of Plant Pathology 132: 133–146.

[4] Figueiredo A, Fortes AM, Ferreira S, Sebastiana M, Choi YH, et al (2008) Transcriptional and metabolic profiling of grape (Vitis vinifera L.) leaves unravel possible innate resistance against pathogenic fungi. Journal of Experimental Botany 59: 3371–3381. 18648103 10.1093/jxb/ern187

[5] Le Henanff G, Heitz T, Mestre P, Mutterer J, Walter B, et al (2009) Characterization of Vitis vinifera NPR1 homologs involved in the regulation of Pathogenesis-Related gene expression. BMC Plant Biology 9: 54. 19432948 10.1186/1471-2229-9-54 PMC2686700

[6] Bustin S (2000) Absolute quantification of mRNA using real-time reverse transcription polymerase chain reaction assays. Journal of Molecular Endocrinology 25: 169–193. 11013345 10.1677/jme.0.0250169

[7] Vandesompele J, De Preter K, Pattyn F, Poppe B, Van Roy N, et al (2002) Accurate normalization of real-time quantitative RT-PCR data by geometric averaging of multiple internal control genes. Genome Biol 3: RESEARCH0034. 12184808 10.1186/gb-2002-3-7-research0034 PMC126239

[8] Artico S, Nardeli S, Brilhante O, Grossi-de-Sa M, Alves-Ferreira M (2010) Identification and evaluation of new reference genes in Gossypium hirsutum for accurate normalization of real-time quantitative RT-PCR data. BMC Plant Biology 10: 49. 20302670 10.1186/1471-2229-10-49 PMC2923523

[9] Derveaux S, Vandesompele J, Hellemans J (2010) How to do successful gene expression analysis using real-time PCR. Methods 50: 227–230. 19969088 10.1016/j.ymeth.2009.11.001

[10] Chao WS, Doğramaci M, Foley ME, Horvath DP, Anderson JV (2012) Selection and Validation of Endogenous Reference Genes for qRT-PCR Analysis in Leafy Spurge (Euphorbia esula). PLoS ONE 7: e42839. 22916167 10.1371/journal.pone.0042839 PMC3419244

[11] Gamm M, Héloir M-C, Kelloniemi J, Poinssot B, Wendehenne D, et al (2011) Identification of reference genes suitable for qRT-PCR in grapevine and application for the study of the expression of genes involved in pterostilbene synthesis. Molecular Genetics and Genomics 285: 273–285. 21340517 10.1007/s00438-011-0607-2

[12] Gantasala N, Papolu P, Thakur P, Kamaraju D, Sreevathsa R, et al (2013) Selection and validation of reference genes for quantitative gene expression studies by real-time PCR in eggplant (Solanum melongena L). BMC Research Notes 6: 312. 23919495 10.1186/1756-0500-6-312 PMC3750715

[13] Goulao L, Fortunato A, Ramalho JC (2012) Selection of Reference Genes for Normalizing Quantitative Real-Time PCR Gene Expression Data with Multiple Variables in Coffea spp. Plant Molecular Biology Reporter 30: 741–759.

[14] Reddy DS, Bhatnagar-Mathur P, Cindhuri KS, Sharma KK (2013) Evaluation and Validation of Reference Genes for Normalization of Quantitative Real-Time PCR Based Gene Expression Studies in Peanut. PLoS ONE 8: e78555. 24167633 10.1371/journal.pone.0078555 PMC3805511

[15] Zhu J, Zhang L, Li W, Han S, Yang W, et al (2013) Reference Gene Selection for Quantitative Real-time PCR Normalization in Caragana intermedia under Different Abiotic Stress Conditions. PLoS ONE 8: e53196. 23301042 10.1371/journal.pone.0053196 PMC3534648

[16] Andersen CL, Jensen JL, Ørntoft TF (2004) Normalization of Real-Time Quantitative Reverse Transcription-PCR Data: A Model-Based Variance Estimation Approach to Identify Genes Suited for Normalization, Applied to Bladder and Colon Cancer Data Sets. Cancer Research 64: 5245–5250. 15289330 10.1158/0008-5472.CAN-04-0496

[17] Pfaffl M, Tichopad A, Prgomet C, Neuvians T (2004) Determination of stable housekeeping genes, differentially regulated target genes and sample integrity: BestKeeper – Excel-based tool using pair-wise correlations. Biotechnology Letters 26: 509–515. 15127793 10.1023/b:bile.0000019559.84305.47

[18] Reid K, Olsson N, Schlosser J, Peng F, Lund S (2006) An optimized grapevine RNA isolation procedure and statistical determination of reference genes for real-time RT-PCR during berry development. BMC Plant Biology 6: 1–11. 17105665 10.1186/1471-2229-6-27 PMC1654153

[19] Coito J, Rocheta M, Carvalho L, Amâncio S (2012) Microarray-based uncovering reference genes for quantitative real time PCR in grapevine under abiotic stress. BMC Research Notes 5: 1–12. 22564373 10.1186/1756-0500-5-220 PMC3837474

[20] Gonzalez-Aguero M, Garcia-Rojas M, Di Genova A, Correa J, Maass A, et al (2013) Identification of two putative reference genes from grapevine suitable for gene expression analysis in berry and related tissues derived from RNA-Seq data. BMC Genomics 14: 878. 24330674 10.1186/1471-2164-14-878 PMC3878734

[21] Monteiro F, Sebastiana M, Pais MS, Figueiredo A (2013) Reference Gene Selection and Validation for the Early Responses to Downy Mildew Infection in Susceptible and Resistant Vitis vinifera Cultivars. PLoS ONE 8: e72998. 24023800 10.1371/journal.pone.0072998 PMC3762845

[22] Selim M, Legay S, Berkelmann-Löhnertz B, Langen G, Kogel KH, et al (2012) Identification of suitable reference genes for real-time RT-PCR normalization in the grapevine-downy mildew pathosystem. Plant Cell Reports 31: 205–216. 22006104 10.1007/s00299-011-1156-1

[23] Borges AF, Ferreira RB, Monteiro S (2013) Transcriptomic changes following the compatible interaction Vitis vinifera–Erysiphe necator. Paving the way towards an enantioselective role in plant defence modulation. Plant Physiology and Biochemistry 68: 71–80. 23639450 10.1016/j.plaphy.2013.03.024

[24] Logemann E, Hahlbrock K (2002) Crosstalk among stress responses in plants: Pathogen defense overrides UV protection through an inversely regulated ACE/ACE type of light-responsive gene promoter unit. Proceedings of the National Academy of Sciences of the United States of America 99: 2428–2432. 11842215 10.1073/pnas.042692199 PMC122381

[25] Reymond P, Weber H, Damond M, Farmer EE (2000) Differential Gene Expression in Response to Mechanical Wounding and Insect Feeding in Arabidopsis. The Plant Cell Online 12: 707–719. 10.1105/tpc.12.5.707 PMC139922 10810145

[26] Cheong YH, Chang H-S, Gupta R, Wang X, Zhu T, et al (2002) Transcriptional Profiling Reveals Novel Interactions between Wounding, Pathogen, Abiotic Stress, and Hormonal Responses in Arabidopsis. Plant Physiology 129: 661–677. 12068110 10.1104/pp.002857 PMC161692

[27] Leon J, Rojo E, Sanchez-Serrano JJ (2001) Wound signalling in plants. Journal of Experimental Botany 52: 1–9. 10.1093/jexbot/52.354.1 11181708

[28] Kunz BA, Cahill DM, Mohr PG, Osmond MJ, Vonarx EJ (2006) Plant Responses to UV Radiation and Links to Pathogen Resistance. In: Kwang WJ, editor. International Review of Cytology: Academic Press. 1–40. 10.1016/S0074-7696(06)55001-6 17178464

[29] Pezet R, Perret C, Jean-Denis JB, Tabacchi R, Gindro K, et al (2003) δ-Viniferin, a Resveratrol Dehydrodimer: One of the Major Stilbenes Synthesized by Stressed Grapevine Leaves. Journal of Agricultural and Food Chemistry 51: 5488–5492. 12926902 10.1021/jf030227o

[30] Bonomelli A, Mercier L, Franchel J, Baillieul F, Benizri E, et al (2004) Response of Grapevine Defenses to UV – C Exposure. American Journal of Enology and Viticulture 55: 51–59.

[31] Fischer M, Kassemeyer HH (2003) Fungi associated with Esca disease of grapevine in Germany. Vitis 42: 109–116.

[32] Gaforio L, Garcia-Munoz S, Cabello F, Munoz-Organero G (2011) Evaluation of susceptibility to powdery mildew (Erysiphe necator) in Vitis vinifera varieties. Vitis 50: 123–126.

[33] Bertsch C, Ramírez-Suero M, Magnin-Robert M, Larignon P, Chong J, et al (2013) Grapevine trunk diseases: complex and still poorly understood. Plant Pathology 62: 243–265.

[34] Gambino G, Perrone I, Gribaudo I (2008) A Rapid and effective method for RNA extraction from different tissues of grapevine and other woody plants. Phytochemical Analysis 19: 520–525. 18618437 10.1002/pca.1078

[35] Pihur V, Datta S, Datta S (2009) RankAggreg, an R package for weighted rank aggregation. BMC Bioinformatics 10: 62. 19228411 10.1186/1471-2105-10-62 PMC2669484

[36] Mallona I, Lischewski S, Weiss J, Hause B, Egea-Cortines M (2010) Validation of reference genes for quantitative real-time PCR during leaf and flower development in Petunia hybrida. BMC Plant Biol 10: 4. 20056000 10.1186/1471-2229-10-4 PMC2827423

[37] Nicot N, Hausman J-F, Hoffmann L, Evers D (2005) Housekeeping gene selection for real-time RT-PCR normalization in potato during biotic and abiotic stress. Journal of Experimental Botany 56: 2907–2914. 16188960 10.1093/jxb/eri285

[38] Jain M, Nijhawan A, Tyagi AK, Khurana JP (2006) Validation of housekeeping genes as internal control for studying gene expression in rice by quantitative real-time PCR. Biochemical and Biophysical Research Communications 345: 646–651. 16690022 10.1016/j.bbrc.2006.04.140

[39] Lovdal T, Lillo C (2009) Reference gene selection for quantitative real-time PCR normalization in tomato subjected to nitrogen, cold, and light stress. Anal Biochem 387: 238–242. 19454243 10.1016/j.ab.2009.01.024

[40] Xu M, Zhang B, Su X, Zhang S, Huang M (2011) Reference gene selection for quantitative real-time polymerase chain reaction in Populus. Anal Biochem 408: 337–339. 20816740 10.1016/j.ab.2010.08.044

[41] Wu G, Zhang L, Wu Y, Cao Y, Lu C (2010) Comparison of five endogenous reference genes for specific PCR detection and quantification of Brassica napus. J Agric Food Chem 58: 2812–2817. 20143854 10.1021/jf904255b

[42] Gjetting T, Carver TL, Skot L, Lyngkjaer MF (2004) Differential gene expression in individual papilla-resistant and powdery mildew-infected barley epidermal cells. Mol Plant Microbe Interact 17: 729–738. 15242167 10.1094/MPMI.2004.17.7.729

[43] Peccoud J, Jacob C (1998) Statistical Estimations of PCR Amplification Rates. In: Ferré F, editor. Gene Quantification: Birkhäuser Boston. 111–128.

[44] Ramakers C, Ruijter JM, Deprez RHL, Moorman AFM (2003) Assumption-free analysis of quantitative real-time polymerase chain reaction (PCR) data. Neuroscience Letters 339: 62–66. 12618301 10.1016/s0304-3940(02)01423-4

[45] Ruijter JM, Ramakers C, Hoogaars WM, Karlen Y, Bakker O, et al (2009) Amplification efficiency: linking baseline and bias in the analysis of quantitative PCR data. Nucleic Acids Res 37: e45. 19237396 10.1093/nar/gkp045 PMC2665230

[46] Castro-Quezada P, Aarrouf J, Claverie M, Favery B, Mugniéry D, et al (2013) Identification of Reference Genes for Normalizing RNA Expression in Potato Roots Infected with Cyst Nematodes. Plant Molecular Biology Reporter 31: 936–945.

[47] Exposito-Rodriguez M, Borges A, Borges-Perez A, Perez J (2008) Selection of internal control genes for quantitative real-time RT-PCR studies during tomato development process. BMC Plant Biology 8: 131. 19102748 10.1186/1471-2229-8-131 PMC2629474

[48] Figueiredo A, Loureiro A, Batista D, Monteiro F, Varzea V, et al (2013) Validation of reference genes for normalization of qPCR gene expression data from Coffea spp. hypocotyls inoculated with Colletotrichum kahawae. BMC Research Notes 6: 388. 24073624 10.1186/1756-0500-6-388 PMC3849654

[49] Xie F, Xiao P, Chen D, Xu L, Zhang B (2012) miRDeepFinder: a miRNA analysis tool for deep sequencing of plant small RNAs. Plant Molecular Biology 80: 75–84. 10.1007/s11103-012-9885-2 22290409

[50] MacDonald MJ, D'Cunha GB (2007) A modern view of phenylalanine ammonia lyase. Biochemistry and Cell Biology 85: 273–282. 17612622 10.1139/o07-018

[51] Mellway RD, Tran LT, Prouse MB, Campbell MM, Constabel CP (2009) The wound-, pathogen-, and ultraviolet B-responsive MYB134 gene encodes an R2R3 MYB transcription factor that regulates proanthocyanidin synthesis in poplar. Plant Physiol 150: 924–941. 19395405 10.1104/pp.109.139071 PMC2689947

